# Ground Cover Rice Production System Affects Soil Water, Nitrogen Dynamics and Crop Growth Differentially with or without Climate Stress

**DOI:** 10.3390/plants12223866

**Published:** 2023-11-15

**Authors:** Jian Ren, Puyu Feng, William D. Batchelor, Kelin Hu, Haitao Liu, Shihua Lv

**Affiliations:** 1 College of Land Science and Technology, China Agricultural University, Key Laboratory of Arable Land Conservation (North China), Ministry of Agriculture and Rural Affairs, Beijing 100193, China; renjian@cau.edu.cn (J.R.); fengpuyu@cau.edu.cn (P.F.); 2 Biosystems Engineering Department, Auburn University, Auburn, AL 36849, USA; bbatch@auburn.edu; 3 Institute of Agricultural Resources and Environment, Sichuan Academy of Agricultural Sciences, Chengdu 610066, China; liuht1986@163.com (H.L.); sclush@126.com (S.L.)

**Keywords:** ground cover rice production system, soil water and nitrogen dynamics, rice yield, climate stress, structural equation modeling

## Abstract

The ground cover rice production system (GCRPS) has been proposed as a potential solution to alleviate seasonal drought and early low-temperature stress in hilly mountainous areas; clarifying its impact on crop growth is crucial to enhance rice productivity in these areas. A two-year (2021–2022) field experiment was conducted in the hilly mountains of southwest China to compare the effects of the traditional flooding paddy (Paddy) and GCRPS under three different nitrogen (N) management practices (N1, zero-N fertilizer; N2, 135 kg N ${ha}^{-1}$ as a urea-based fertilizer; and N3, 135 kg N ${ha}^{-1}$ with a 3:2 base-topdressing ratio as urea fertilizer for the Paddy or a 1:1 basal application ratio as urea and manure for GCRPS) on soil water storage, soil mineral N content and crop growth parameters, including plant height, tiller numbers, the leaf area index (LAI), aboveground dry matter (DM) dynamics and crop yield. The results showed that there was a significant difference in rainfall between the two growth periods, with 906 mm and 291 mm in 2021 and 2022, respectively. While GCRPS did not significantly affect soil water storage, soil mineral N content, and plant height, it led to a reduction in partial tiller numbers (1.1% to 31.6%), LAI (0.6% to 20.4%), DM (4.4% to 18.8%), and crop yield (7.4% to 22.0%) in 2021 (wet year) compared to the Paddy. However, in 2022 (dry year), GCRPS led to an increase in tiller numbers (13.7% to 115.4%), LAI (17.3% to 81.0%), DM (9.0% to 62.6%), and crop yield (2.9% to 9.2%) compared to the Paddy. Structural equation modeling indicated that GCRPS significantly affected tiller numbers, plant height, LAI, DM, and productive tiller numbers, which indirectly influenced crop yield by significantly affecting tiller numbers and productive tiller numbers in 2022. Overall, the effects of GCRPS on soil water and N dynamics were not significant. In 2021, with high rainfall, no drought, and no early, low-temperature stress, the GCRPS suppressed crop growth and reduced yield, while in 2022, with drought and early low-temperature stress and low rainfall, the GCRPS promoted crop growth and increased yield, with tiller numbers and productive tiller numbers being the key factors affecting crop yield.

## 1. Introduction

China is among the largest rice producers globally, cultivating an area of 30.5 million ha and producing 220 million tons of grain yield, which corresponds to approximately 19% of the world’s planted area and 30% of global rice yield [1]. Rice plays a critical role in the country’s grain production system, constituting 38% and 45% of the total planted area and grain yield, respectively, of the three major grain crops (rice, wheat, and maize) [2]. Currently, flood irrigation is the primary water management practice used in the rice fields of China [3,4]. However, this method requires a significant amount of irrigation water, resulting in a shortage of water resources and limiting rice production in arid regions [5,6,7]. Rice cultivation in the hilly mountainous regions of China is often influenced by seasonal drought aggravated by inadequate irrigation conditions, leading to a shortage of irrigation water [8,9]. Furthermore, this hilly mountainous region in China often experiences low temperatures in late spring and early summer, which can delay rice planting and shorten the growth period [10]. Meanwhile, with the frequent occurrence of extreme drought in recent years, rice production in this region faces serious challenges [11]. Therefore, developing rice production systems that conserve water and raise temperatures could lead to higher yields and improved sustainability of production in this region.

To increase the yield and sustainability of rice production in these hilly mountainous areas, several water management practices have been developed, including wetting-and-drying irrigation, dry-seeding techniques, and the ground cover rice production system (GCRPS) [12]. GCRPS is a potential water management technique for rice cultivation that aims to avoid standing water in fields during the rice growth period. This system involves covering the soil surface with a thin (5–7 μm) plastic film, which helps to maintain the topsoil water content in a non-saturated condition that is conducive to rice production [13,14]. Due to the mulching technique, GCRPS not only conserves water through reduced soil evaporation [15,16,17] but also raises the soil temperature [18,19], which alleviates early low-temperature stress. This creates favorable conditions that promote crop growth and increase crop yield [20,21]. Moreover, GCRPS effectively mitigates greenhouse gas emissions and the prevalence of weeds and pests in rice fields [22,23,24], which has led to its widespread adoption in hilly mountainous areas of the middle and lower reaches of the Yangtze River [25].

The plastic film cover in GCRPS makes split fertilizer applications impossible; thus, it is necessary to apply all fertilizer at once as a single basal application. This leads to excessive rice growth during the early stages and nitrogen (N) stress during the late growth stages, resulting in reduced crop yield and limiting the potential to increase this yield [26,27]. To address this issue, the use of controlled-release and organic fertilizers as base fertilizers was suggested [28]. However, the cost and promotion of controlled-release fertilizers are challenging, whereas organic fertilizers are less costly and more readily available [29]. Studies have shown that the combined application of inorganic and organic fertilizers can regulate soil N mineralization and provide sufficient N at late growth stages, thereby enhancing crop yield [30].

At present, studies on GCRPS have mainly focused on the impacts of soil water, temperature, nutrients, and crop growth. Most studies have focused on soil mineral N (SMN) content, and the results of the effect of GCRPS are mixed [8,25,31]. Reports on the effect of GCRPS on soil water content do not have direct comparisons with the results of the traditional flooding paddy system, and the differences between them are not very clear. Prior studies have been conducted to determine the impact of GCRPS on crop growth; however, the conclusions are also mixed, with GCRPS either promoting or inhibiting crop growth and increasing or decreasing yields [18,32,33,34]. Consequently, the effects of GCRPS on soil water, N content, and crop growth remain uncertain, and the indirect effect pathways of GCRPS on crop yield are not well defined. Thus, further investigation is needed to understand the effects of GCRPS on soil water, N content, and crop growth.

To further explore the reason that led to different findings, a two-year (2021–2022) field experiment was conducted to investigate the impact of different water and N management practices on soil water content, SMN content, rice plant height, tiller numbers, the leaf area index (LAI) and aboveground dry matter (DM) dynamics during rice growth periods, and crop yield in the hilly mountainous area of southwest China. The objectives of this work were as follows: (i) evaluate the effects of GCRPS on soil water, N dynamics, and crop growth; and (ii) explore the key factors influencing crop yield under GCRPS using structural equation modeling (SEM). These findings are expected to provide insights into the applicability of GCRPS in this region.

## 2. Results

### 2.1. Effect of Ground Cover Rice Production System on the Dynamics of Soil Water Storage

**Figure 1:**
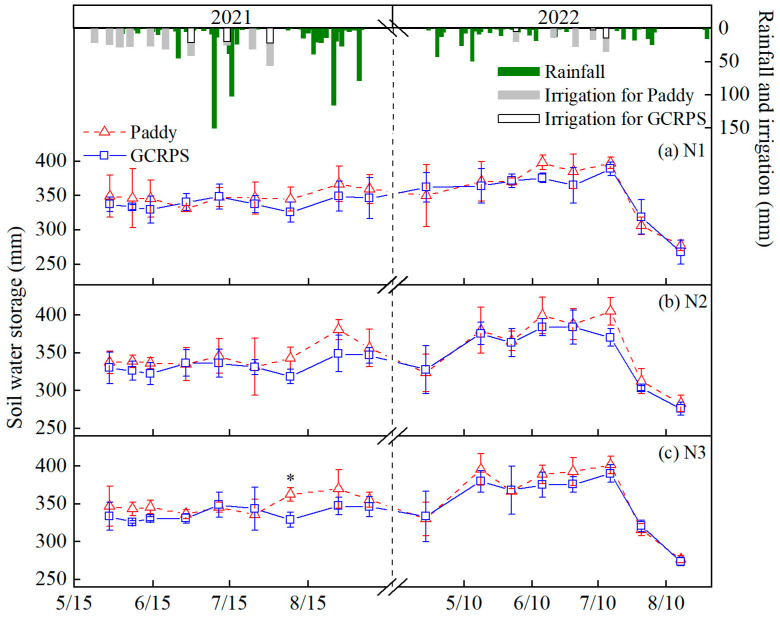
Soil water storage dynamics in the 0–80 cm soil profile under different water and N treatments. * Significant at 0.05 probability level. Vertical bars represent standard error of mean. Paddy, traditional flooding paddy; GCRPS, ground cover rice production system; N1, no fertilizer; N2, urea-based fertilizer; N3, urea, and manure-based fertilizer.

Figure 1 shows the soil water storage (SWS) in the 0–80 cm soil profile under different treatments. In 2021, the SWS ranged from 330 to 380 mm and 312 to 360 mm for both Paddy and GCRPS treatments, respectively, and the SWS for GCRPS treatment was generally lower than the Paddy treatment. Before the maximum tillering stage, rainfall was less, and SWS tended to decrease but did not fluctuate much. From the maximum tillering stage to the panicle initiation stage, there were two major rainstorms, resulting in essentially the same SWS in Paddy and GCRPS treatments, but it did not increase continuously, indicating that the soil had reached saturation during this period. After the panicle initiation stage, SWS tended to increase with frequent rainfall events during this period and then decreased with the pre-harvest drainage of rice fields. In 2022, the fluctuation range of SWS was relatively large for treatments using Paddy (276 to 405 mm) and relatively small for the GCRPS treatment (267 to 390 mm). Since there was heavy rain before the maximum tillering stage, SWS showed a gradual upward trend and then continued to decrease with decreasing rainfall. Between the stage of maximum tillering and panicle initiation, both rainfall and irrigation were less but relatively and evenly distributed, resulting in small fluctuations in SWS. However, after the panicle initiation stage, rainfall was low, and drought occurred from the end of July to the end of August, leading to a significant decrease in SWS for both water treatments.

Overall, the differences in SWS for different water and N management practices were not significant, while the differences in SWS for different years were more obvious, with the highest values of 380 mm and 405 mm over the two years, respectively, and it was higher in 2022 due to autumn plowing before the experiment in 2022, which increased the capacity of SWS. Secondly, SWS presented a gradually decreasing trend before the maximum tillering stage in 2021, while it showed a rising and then decreasing trend in 2022, which was due to the higher temperature during this period in 2021, as shown in the ninth figure resulting in more water loss via evaporation. Additionally, compared with 2022, SWS was less affected by rice field drainage in the pre-harvest of 2021 due to frequent rainfall and two storms that occurred during this period. In 2022, very little rainfall and prolonged high temperatures in the late growth period 2022 resulted in a more obvious reduction in SWS after rice field drainage.

### 2.2. Effect of Ground Cover Rice Production System on the Dynamics of Soil Mineral N

**Figure 2:**
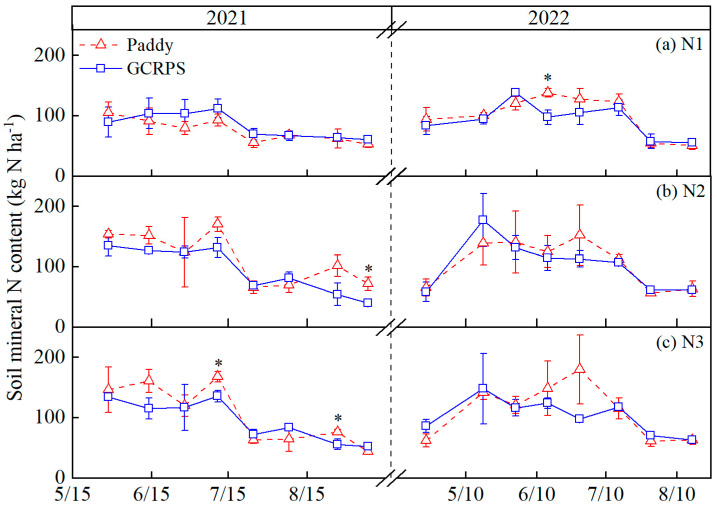
Soil mineral N dynamics in the 0–80 cm soil profile under different water and N treatments. * Significant at 0.05 probability level. Vertical bars represent standard error of mean. Paddy, traditional flooding paddy; GCRPS, ground cover rice production system; N1, no fertilizer; N2, urea-based fertilizer; N3, urea, and manure-based fertilizer.

Figure 2 shows SMN in the 0–80 cm soil profile under different treatments. In general, there was a gradual decrease in SMN with crop growth for all treatments. Before the maximum tillering stage in 2021, the nutrient demand of the crop was low, resulting in high SMN levels (120 to 170 kg N ${ha}^{-1}$) and small overall variations in the SMN content. Except for N1, the order of SMN content for N2 and N3 was Paddy > GCRPS, probably due to the heavy rainfall during this period, which caused the soil to be in an anaerobic state for a short period and reduced soil nitrification, thus affecting soil N transformation. After the panicle initiation stage, SMN content gradually decreased, which was due to crop N uptake during this period and the high N runoff loss due to frequent rainfall. SMN reached its lowest level when the field was drained and dried for harvest.

Before the mid-tillering stage in 2022, the soil mineral N content increased rapidly from about 60 to 170 kg N ${ha}^{-1}$, with the exception of N1. GCRPS had a higher SMN content than Paddy for N2 and N3 due to lower rainfall and early low-temperature stress, which accelerated soil mineralization and nitrification. Between the mid-tillering stage and the panicle initiation stage, the SMN content for GCRPS was less than Paddy, which could be caused by topdressing for the Paddy. After the panicle initiation stage, SMN gradually decreased from about 120 kg N ${ha}^{-1}$ to 50 kg N ${ha}^{-1}$ due to high temperatures and less rainfall, and there was no significant difference between Paddy and GCRPS.

Overall, there was no significant difference between paddy rice and GCRPS management on SMN. However, N fertilizer application had a significant effect on the SMN content. N fertilizer application increased the SMN content before the maximum tiller stage during the two-year experiment. SMN in treatments N2 and N3 were higher than in treatment N1 by 17.3% to 84.4% and 10.8% to 81.6%, respectively, in 2021 and by 7.3% to 88.1% and 7.9% to 56.8%, respectively, in 2022, while there was no obvious difference during the remaining growing season. No obvious difference was found in the SMN content of treatments N2 and N3.

### 2.3. Effect of Ground Cover Rice Production System on Crop Growth

#### 2.3.1. Plant Height Dynamics

**Figure 3:**
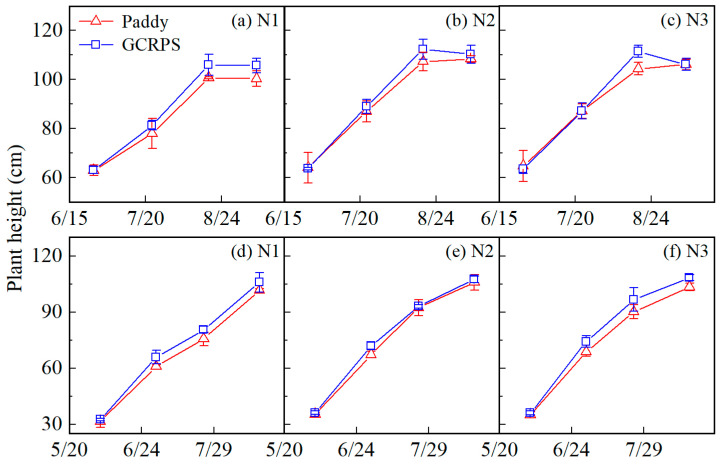
Plant height under different water and N treatments in 2021 (**a**–**c**) and 2022 (**d**–**f**). Vertical bars represent standard error of mean. Paddy, traditional flooding paddy; GCRPS, ground cover rice production system; N1, no fertilizer; N2, urea-based fertilizer; N3, urea, and manure-based fertilizer.

Figure 3 shows the measured plant height for different treatments. In 2021, plant height gradually increased before the grain-filling stage but then remained basically unchanged for all treatments except for treatment GCRPS_N3, where there was a slight late-season decrease in plant height. The ranges of plant height under Paddy and GCRPS treatment were from 63 to 108 cm and 63 to 112 cm, respectively, which were fairly consistent and not significantly different. In 2022, plant height gradually increased through the reproductive stages, and the ranges for Paddy and GCRPS treatment were from 32 to 106 cm and 32 to 108 cm, respectively, which were essentially consistent and not significantly different.

#### 2.3.2. Tiller Dynamics

**Figure 4:**
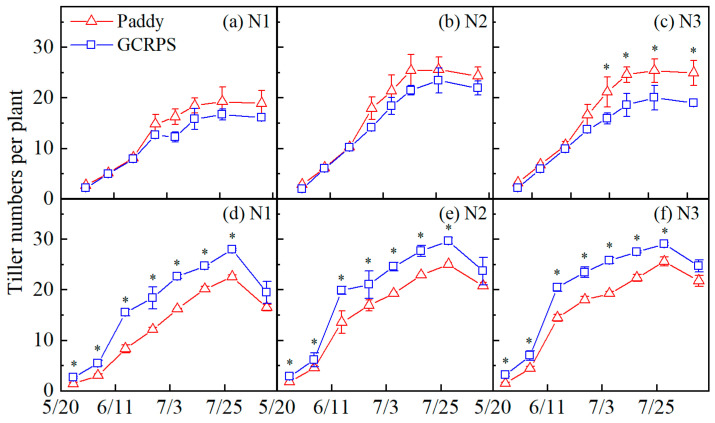
Tiller numbers under different water and N treatments in 2021 (**a**–**c**) and 2022 (**d**–**f**). * Significant at 0.05 probability level. Vertical bars represent standard error of mean. Paddy, traditional flooding paddy; GCRPS, ground cover rice production system; N1, no fertilizer; N2, urea-based fertilizer; N3, urea, and manure-based fertilizer.

Figure 4 shows the rice tillering dynamics of different treatments. The rice tillering stage lasted for about 80 days each year, starting in late May. Tiller numbers peaked in late July and gradually decreased thereafter, with a larger decrease in 2022 due to high temperatures and drought. In 2021, tiller numbers were not significantly different among different water treatments from the early tillering stage to the middle tillering stage, while differences occurred from the maximum tillering stage to the grain filling stage. GCRPS exhibited lower tiller numbers compared to the Paddy for all N treatments (1.1% to 31.6%). Notably, treatment N3 demonstrated significant differences. However, in 2022, GCRPS displayed higher tiller numbers than Paddy for all N treatments (13.7% to 115.4%). These differences were significant from the early tillering to the grain filling stage.

The application of N fertilizer led to an increase in tiller numbers throughout the growth period. Treatments N2 and N3 exhibited higher tiller numbers compared to treatment N1, with an increase ranging from 7.9% to 50.0% in 2021 and 4.0% to 74.5% in 2022, respectively. However, there were no significant differences observed between treatments N2 and N3 in either year.

#### 2.3.3. Leaf Area Index Dynamics

**Figure 5:**
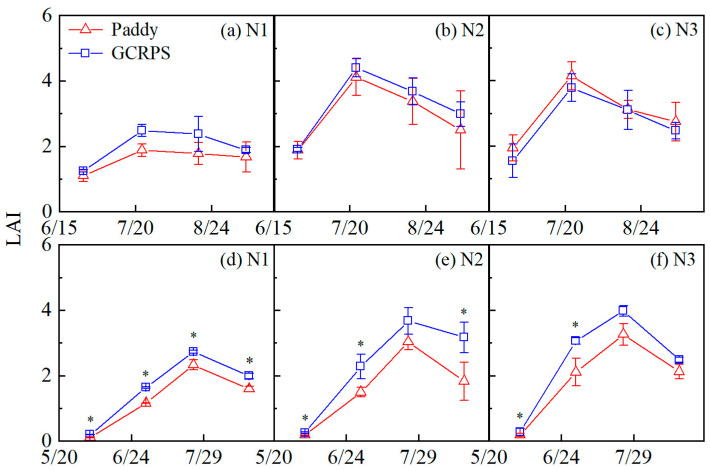
Leaf area index under different water and N treatments in 2021 (**a**–**c**) and 2022 (**d**–**f**). * Significant at 0.05 probability level. Vertical bars represent standard error of mean. Paddy, traditional flooding paddy; GCRPS, ground cover rice production system; N1, no fertilizer; N2, urea-based fertilizer; N3, urea, and manure-based fertilizer.

The LAI of different treatments is shown in Figure 5. LAI showed an overall trend of increasing until the reproductive stages, then decreasing until harvest in all treatments. For different water treatments in 2021, GCRPS had higher LAI values (1.1% to 34.0%) than Paddy for both the N1 and N2 treatments. However, for treatment N3, GCRPS had lower LAI values throughout the season than Paddy (0.6% to 20.4%), although these differences were not significant. In 2022, GCRPS had higher LAI values (17.3% to 81.0%) than Paddy for all N treatments. Significant differences in LAI were found among different water treatments, with significant differences in treatment N1 throughout the season. Significant differences in treatment N2 at the maximum tillering, panicle initiation, and maturity stages were found, as well as significant differences in treatment N3 at the maximum tillering and panicle initiation stages.

The application of N fertilizer resulted in an increase in LAI throughout the season. Treatments N2 and N3 had a higher LAI compared to treatment N1, with an increase ranging from 15.0% to 125.5% during 2021 and 2022. However, there were no significant differences observed between treatments N2 and N3 in either year.

#### 2.3.4. Aboveground Dry Matter Dynamics

**Figure 6:**
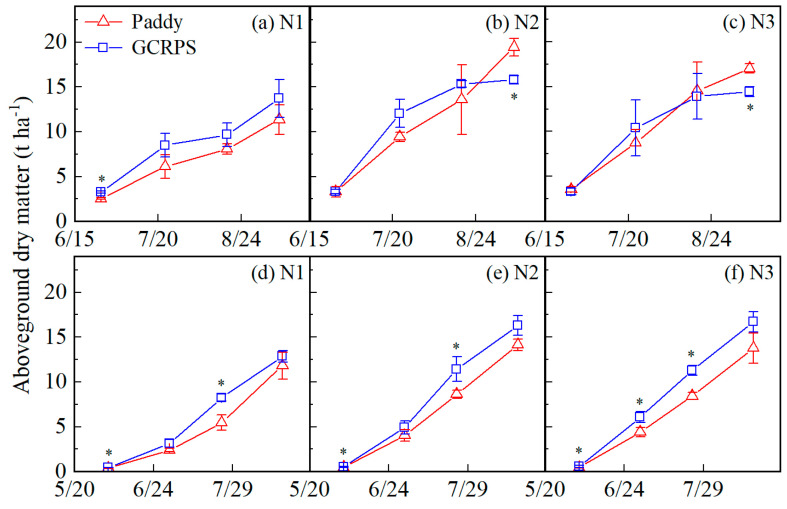
Aboveground dry matter under different water and N treatments in 2021 (**a**–**c**) and 2022 (**d**–**f**). * Significant at 0.05 probability level. Vertical bars represent standard error of mean. Paddy, traditional flooding paddy; GCRPS, ground cover rice production system; N1, no fertilizer; N2, urea-based fertilizer; N3, urea, and manure-based fertilizer.

The DM of different treatments is shown in Figure 6. The overall trend was a gradual increase in DM over the season in both years. In 2021, the DM for treatment N1 was always higher for GCRPS than for the Paddy (19.6% to 38.8%), and there was a significant difference at the maximum tillering stage. Treatment with GCRPS had higher DM between the maximum tillering to grain filling stage and panicle initiation stage for treatments N2 and N3 (0.6% to 27.7%). However, DM in GCRPS decreased below that of the Paddy at the maturity stage, and the stages of maximum tillering, grain filling, and maturity for N2 and N3 treatments (4.4% to 18.8%), and significant differences were only observed at the maturity stage. In 2022, the DM for GCRPS was always higher than the Paddy for all N treatments (9.0% to 62.6%). Significant differences were observed at the maximum tillering and grain-filling stages for treatments N1 and N2, while for treatment N3, significant differences were observed before the maturity stage.

N fertilizer rates increased the DM throughout the season, with treatments N2 and N3 having a 1.5% to 81.1% higher DM than treatment N1 in 2021. There was no significant difference observed between treatments N2 and N3. Similarly, in 2022, with treatments N2 and N3 having a 19.9% to 69.0% and 16.6% to 93.3% higher DM than treatment N1, respectively, treatment N3 was slightly higher than treatment N2.

#### 2.3.5. Crop Yield

**Figure 7:**
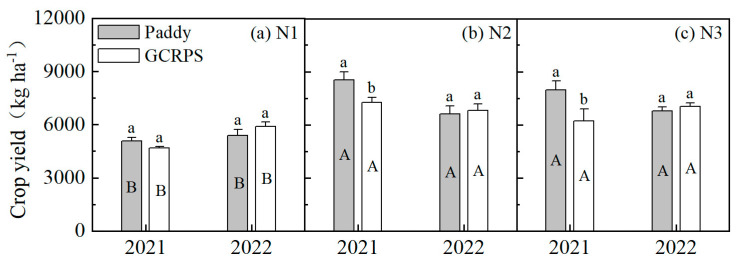
Crop yield under different water and N treatments. Bars labeled with the same capital letters and lowercase letters show no significant differences (*p* < 0.05) between different N treatments and different water treatments, respectively. Paddy, traditional flooding paddy; GCRPS, ground cover rice production system; N1, no fertilizer; N2, urea-based fertilizer; N3, urea and manure-based fertilizer.

The crop yield for different treatments is shown in Figure 7. Yields ranged from 4600 to 8905 kg ${ha}^{-1}$ in 2021 and 4922 to 7332 kg ${ha}^{-1}$ in 2022. The highest yield in 2022 was significantly lower than that in 2021 due to the extremely high temperatures for about one month during the late growth period in this year that affected it. Compared to treatment with Paddy, treatment with GCRPS had a lower rice yield in 2021 for all N treatments (7.4% to 22.0%), with significant differences for treatments N2 and N3. In 2022, GCRPS treatment had a higher rice yield for all N treatments (2.9% to 9.2%). However, the differences were not statistically significant.

Regarding the different N treatments, the application of N fertilizer rates led to an increase in rice yield. Treatments N2 and N3 exhibited a rice yield increase ranging from 16.0% to 68.8% in 2021 and 2022 compared to treatment N1. These results clearly demonstrate that treatments N2 and N3 significantly outperformed treatment N1, while there was no significant difference observed between treatments N2 and N3.

There was some variability in yield for different years. Compared with the crop yield in 2021, the crop yield of treatment N1 in 2022 showed an increasing trend, whereas treatment N2 exhibited a decreasing trend. For treatment N3, the Paddy treatment displayed a decreasing trend, while treatment with GCRPS exhibited an increasing trend.

The results of the analysis of variance (ANOVA) are presented in Table 1. The crop yield was significantly influenced by both water and N management practices but not by year. Furthermore, a significant interaction was observed between the year and both water and N management practices.

### 2.4. Direct and Indirect Effects of Ground Cover Rice Production System on Soil Water and N Dynamics and Crop Growth

**Figure 8:**
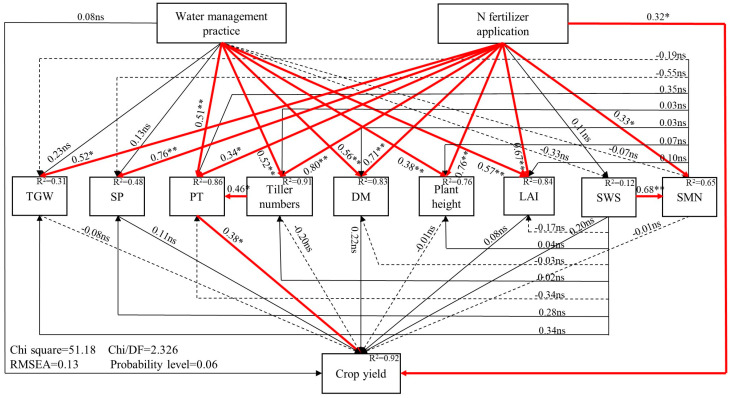
The structural equation modeling linking water management practices, N fertilizer application, soil water storage, soil mineral N content and crop growth in 2022. ns, not significant. * or ** significant at 0.05 or 0.01 probability level, respectively. Red lines represent significant effects. The solid and dashed lines represent positive and negative effects, respectively.

The SEM linking water management practices, N fertilizer application, soil water and N dynamics, and crop growth is shown in Figure 8. Taking 2022 as an example, the SEM considered all possible pathways affecting crop yield throughout the season. Since there was little difference in SWS, the SMN content and crop growth between treatments N2 and N3 and N management practices were not considered in this study, and N fertilizer application rate was used as a surrogate. The variables in Figure 8 jointly explain 92% of the variance in crop yield. First, compared with the Paddy, GCRPS significantly affected plant height, tiller numbers, the LAI, DM, and the number of productive tillers(PT), while the effects on thousand-grain weight (TGW), the number of spikelets per panicle (SP), and the SWS and SMN content were not significant. Meanwhile, N fertilizer application significantly affected all crop biological indicators and the SMN content, while the effect on SWS was not significant. Second, only PT significantly affected crop yield among crop biological indicators and the soil water and N content. Tiller numbers significantly affected PT among the crop biological indicators. Third, GCRPS did not affect crop yield directly but mainly did so indirectly by affecting tiller numbers and PT. N fertilizer application significantly affected crop yield and also indirectly affected crop yield by significantly affecting tiller numbers and PT.

Furthermore, the effects of GCRPS on SWS and SMN content were negative, but these effects were not significant. SWS significantly affected the SMN content. Neither the SWS nor SMN content had a significant effect on crop biological indicators.

## 3. Discussion

### 3.1. Effects of Ground Cover Rice Production System on Soil Water and N Dynamics

**Figure 9:**
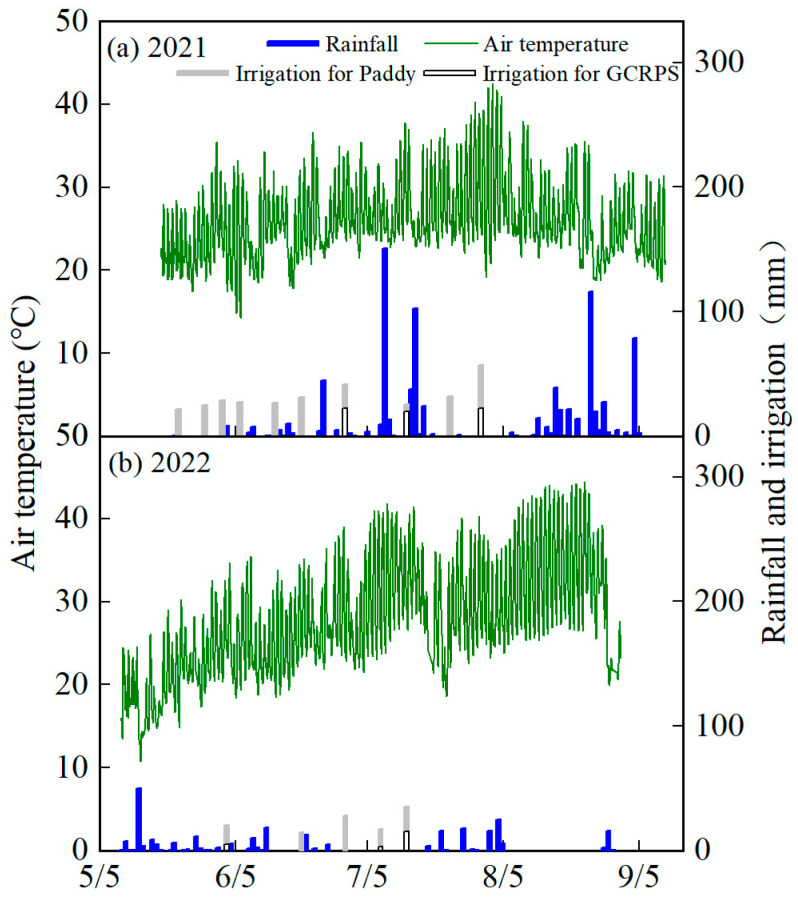
Air temperature, daily rainfall and irrigation at the experimental site in 2021 and 2022.

Soil water status, a necessary aspect of rice production, was mainly influenced by factors such as water management practices and climatic conditions [35]. Compared with the Paddy, Cheng et al. [36] found that GCRPS reduced the soil water content in the hilly areas of Zhejiang Province, China. However, Dong et al. [34] showed that the effect of GCRPS on the soil water content was not significant in our study region. Zhao et al. [37] concluded that although GCRPS has less irrigation, evaporation through the soil’s surface can condense into droplets and return the evaporated water to the soil when blocked by the film, resulting in only a little difference in the soil water content between GCRPS and Paddy. Our results indicate that GCRPS reduced SWS, but the overall effect was not significant compared with the Paddy (Figure 1), which is consistent with the above studies. However, the effect of the year on SWS was more obvious than that of water management practices, which could be related to the large differences in meteorological factors between years. Our results indicate that the overall fluctuation in SWS was small due to the absence of high air temperatures despite the high rainfall during the growth period of 2021. In 2022, although the rainfall before the panicle initiation period was low, there was no high-temperature period, and SWS also fluctuated a little, while the high air temperatures and sparse rainfall after the panicle initiation period (Figure 9) led to a rapid decrease in SWS (Figure 1).

The SMN content represents the level of soil N supply and is mainly influenced by factors such as water and N management practices [38]. Many studies have reported that compared with Paddy, GCRPS increased the soil temperature, accelerated soil N mineralization, and reduced N leaching losses, thus significantly increasing SMN content [39,40,41]. It has also been considered that GCRPS promotes crop growth based on increased SMN, thus increasing crop N demand, resulting in insignificant or even significantly reduced effects of GCRPS on the soil N content [25,31,36,42]. In our study, GCRPS significantly reduced the SMN content only in a few periods during the two years, with no significant differences during the remaining periods (Figure 2), which is similar to the results of previous studies. However, the effect of N fertilizer application on the SMN content was more pronounced compared with water management practices. Previous studies showed that different water management practices had little effect on the SMN content, while N fertilizer application significantly increased the SMN content [25,43]. Our results indicate that N fertilizer application increased the SMN content at the early and middle growth stages (Figure 2), which is also consistent with the above studies.

In addition, previous studies have considered how soil water movement directly influences SMN transport and transformation processes [44,45], showing that there is a relationship between the SMN content and SWS. Therefore, in our study, the relationship between soil water and N was established based on the relationship between GCRPS, soil water, and N. The results of SEM indicated that the SMN content was directly and significantly influenced by SWS (Figure 8), which is in agreement with the results of previous studies.

### 3.2. Effects of Ground Cover Rice Production System on Crop Yield and Biological Indicators

The GCRPS changed the flooding environment for rice growth and affected the soil water and temperature conditions, which, in turn, affected crop growth. The results of previous studies on the effects of GCRPS on crop yield and biological indicators were different, which could be related to meteorological conditions such as rainfall and temperature in the experimental area. Some studies have indicated that GCRPS can effectively mitigate the threats posed by seasonal drought and early low-temperature stress [46] and has greater potential for development in rice-cultivated areas in hilly mountainous areas, with greater advantages in improving rice tillering, LAI, DM, and crop yield [18,47]. Nevertheless, there were also reports where the yield and biological indicators of GCRPS were equal to or even significantly lower than those of the Paddy, and these reports were from subtropical coastal areas, which are associated with excessive rainfall and high-temperature in the early growth period, where both water and temperature conditions are not limiting for crop growth [48,49,50].

In 2021, the rainfall in our study area was 1268 mm, which was higher than the multi-year average rainfall of 966 mm, and rainfall during the rice growth period reached 906 mm (Figure 9), which was higher than the average rainfall during the growth period of 715 mm over the past 15 years [34], so there was no seasonal drought in this year. The temperature in the early growth period was higher than 14 °C during this year (Figure 9), which was within the suitable temperature range for rice growth [32], and there was no early low-temperature stress. Our results for this year show that GCRPS reduced the tiller numbers, LAI, and DM to some extent and reduced yield by 7.4% to 22.0% compared with the Paddy (Figure 7), which is consistent with the results of previous studies [48,49,50]. This is because continuous heavy rainfall resulted in the persistent flooding of GCRPS, which reduced soil air permeability, inhibited soil microbial activity, and led to the increased toxicity of soil-reducing substances [33]. GCRPS also failed to increase soil temperature or even cool it [51], while higher temperatures in the early growth period reduced the soil warming benefit of mulching [47], which, in turn, suppressed rice tillering and led to a reduction in rice yield (Figure 4). Nevertheless, in 2022, rainfall during the growth period was only 265 mm, which was significantly lower than the previous years, and irrigation was also significantly lower than in 2021, as shown in the forth table. Moreover, a longer period of high temperatures was experienced from July to August (Figure 9), in which the number of days with temperatures above 40 °C reached 27 days, and there was no precipitation during this period. Therefore, there was a significant seasonal drought during this year. In addition, there was early low-temperature stress with a 2.2 °C decrease in temperature, which monitored early low temperatures below 14 °C in this year compared with 2021 (Figure 9) [32]. Compared with the Paddy, GCRPS significantly improved tiller numbers, LAI, and DM and increased yield by 2.9% to 9.2% in this year (Figure 7). Excessive rainfall during the growth period and higher temperatures in the early growth period can adversely affect crop growth under GCRPS.

Since rice tillering was a major factor influencing PT, which directly determined crop yield [30], the correlation analysis between the tiller numbers and cumulative rainfall during the tillering period for different water management practices (Table 2) revealed that there was no significant correlation between rice tiller numbers and cumulative rainfall during the tillering period in 2021 and a significant correlation in 2022. This indicates that rice tillering in a dry year is directly affected by rainfall while tillering in a wet year is not affected by rainfall, which is consistent with the results of a previous study [34]. Meanwhile, the tiller numbers in 2022 were significantly higher in the GCRPS than in the Paddy (Figure 4), so it can be further speculated that GCRPS had a better effect in increasing the yield in the dry year.

The results of previous studies in our region showed that a crop yield of 8100 kg ${ha}^{-1}$ to 9500 kg ${ha}^{-1}$ could be achieved in GCRPS under normal and dry years and similar field management with N fertilizer application [52,53,54,55,56]. The rainfall frequency in our study was 4.9% in 2021 [57], a typical and exceptionally wet year with a significant light deficit and crop yield of only 5524 kg ${ha}^{-1}$ to 7515 kg ${ha}^{-1}$ in GCRPS, which is significantly lower than other years.

In summary, the results of our study help to further explain why GCRPS is more suitable for water management practices in rice fields under seasonal drought and early low-temperature stress conditions. It is worth noting that during wet years, the drainage of GCRPS should be improved through appropriate field management practices. This prevents GCRPS from being submerged by water layers, thus reducing the risk of yield reductions. Additionally, irrigation amounts should also be timely adjusted.

On the other hand, it is essential to consider the cost and income comparison between GCRPS and Paddy. Jabran et al. [58] found that while using mulch increased the cost of GCRPS by USD 155 per hectare, the cost of irrigation decreased by USD 162 per hectare due to water savings. Additionally, the absence of herbicides or insecticides resulted in a total reduction in costs by 13%. Lv et al. [53] showed that during dry years, paddy rice farmers incurred losses ranging from CN¥ 3000 to 4500 (USD 1 ≈ CN¥ 7.3) per hectare, whereas those under GCRPS produced gains ranging from CN¥ 4500 to 7500 per hectare. In normal years, paddy rice farmers earned a profit of around CN¥ 1500 per hectare, while those under GCRPS made a profit of CN¥ 9000 to 12,000 per hectare. Therefore, there is a large cost-saving and income-generating advantage in GCRPS. If GCRPS can be further improved to reduce yields in wet years, it could become a more economically viable option for the benefit of rice farmers.

With the increasing frequency of extreme weather events in the future, research on the effects of different climatic conditions on crop growth should be continued in the future, and agricultural technology measures should be reasonably adjusted to adapt to the adverse effects of climate change during the rice growth period in order to maintain or improve rice productivity.

### 3.3. Driving Factors of Crop Yield under Ground Cover Rice Production System

Previous studies have tended to determine the drivers of crop yield under GCRPS based on differences in yield components under different water management practices. Tao et al. [18] showed that PT had the largest effect among yield components under different water management practices. Fan et al. [59] and Kang et al. [60] found that GCRPS significantly increased PT and, thus, crop yield compared with the Paddy in our study region, while the effect on the remaining yield components was not significant. Tao et al. [32] reported both a significant yield reduction and an increase in GCRPS, where GCRPS significantly affected PT but not TGW and SP. However, these studies only analyzed the effects of GCRPS on yield components and did not elucidate the internal causal relationships, especially the relationships between water management practices and crop physiological indicators, soil water, N dynamics, and crop yield. In our study, we analyzed the pathways of the effect of GCRPS on crop yield using SEM with dynamic experimental variables based on the effects of GCRPS on soil water, N dynamics, and crop growth, which helped to reveal the driving factors of crop yield under GCRPS [61].

Our study indicated that GCRPS indirectly affected crop yield by significantly affecting PT, while there were no significant effects on the SP and TGW (Figure 8), which confirmed the results of previous studies. In addition, the above studies analyzed the differences in yield components under different water management practices and also analyzed other crop physiological indicators, among which tiller numbers had the largest effect. Fan et al. [59] concluded that tiller numbers could increase PT and, thus, affect crop yield, showing a strong causal relationship between tiller numbers and PT. Our results show that tiller numbers significantly affected PT and, thus, indirectly affected crop yield (Figure 8), which is in agreement with the previous findings. Our results indicate that the direct drivers of crop yield under GCRPS in this region were tiller numbers and PT. Future research on the physiology and mechanism of tiller numbers and PT in GCRPS should be conducted to understand how to promote the formation of tillers and PT to improve rice productivity in this system.

Meanwhile, some studies also determined the drivers of crop yield under GCRPS via differences in the soil water and N content under different water management practices, and the results showed that the differences in soil water and N content were not significant [34,43]. Our results indicate that GCRPS did not significantly affect the SWS and SMN content, nor did it affect crop yield through indirect effects on both (Figure 8), suggesting that the soil water and N content were not drivers of crop yield under GCRPS, mainly because GCRPS increased soil mineral N mineralization, but crop N uptake also increased, resulting in the non-significant effect of GCRPS on soil N content.

## 4. Materials and Methods

### 4.1. Site Description

The field experiment was conducted at an experimental site located in Ziyang City of Sichuan Province (104°34′ E, 30°05′ N). This area experiences a northern subtropical monsoon climate characterized by an average annual air temperature of 16.8 °C and an average annual rainfall of 966 mm. This area also receives a total of 1300 annual sunshine hours (h). This site represented a typical rice-cultivated area in the hilly mountainous region of China, with an elevation of 395 m and a groundwater level of 1 m. The soil is classified as purple soil and is developed from the parent material of the Suining Group. The basic physical and chemical properties of the 0–20 cm soil layer are shown in Table 3.

The rainfall and temperature during the rice growth period at the site from the 2021 and 2022 seasons are shown in Figure 9. In terms of rainfall, obvious differences were found in the amount and temporal distribution of rainfall between the two seasons, with 906 mm of rainfall in the 2021 season, during which a total of five heavy rainstorms (>100 mm) were observed, including two and one heavy rainstorms in the middle and late growth stages, respectively. Rainfall during the 2022 season was 291 mm, which is significantly lower than in 2021, with only one heavy rainfall (>50 mm) after four days of transplanting and moderate and light rainfall during the remainder of the season. The temporal distribution of temperature during the two seasons was significantly different as well, with a mean temperature of 23.7 °C and 21.5 °C during the early growth period (before the maximum tillering stage) and a minimum temperature of 10.8 °C in 2022, which was lower than the temperature (14.2 °C) in 2021.

### 4.2. Experimental Design and Field Management

**Figure 10:**
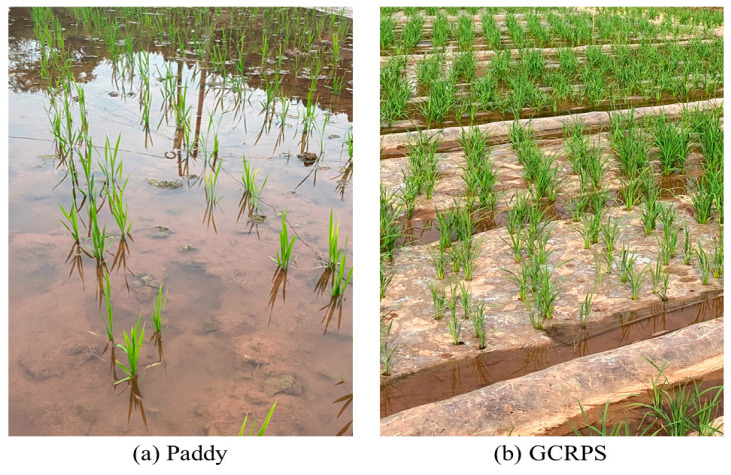
Schematic representation of the field experiment for the traditional flooding paddy (Paddy, (**a**)) and ground cover rice production system (GCRPS, (**b**)).

A two-year field experiment was conducted at the experimental station from May 2021 to September 2022 in a randomized block design. The two water treatments were as follows: (1) Paddy, the traditional flooding paddy, was produced according to the traditional water management practice of local farmers, without a raised bed or plastic mulch, maintaining a 1–5 cm layer of water in the field from transplanting until two weeks before harvest; and (2) GCRPS which was implemented including ridges, plastic mulch, and furrow irrigation. Each experimental block consisted of two raised beds measuring 1.5 m × 9.6 m in area, which were deliberately left without a visible water layer. The furrows surrounding and between the raised beds were flooded with water starting from transplant until two weeks prior to harvest. The soil water content was maintained at 70% to 100% of the saturation water content throughout the rice growth period. A schematic representation of the two water treatments described above is depicted in Figure 10.

Three N treatments were implemented as follows: (1) N1: zero-N fertilizer; (2) N2: 135 kg N ${ha}^{-1}$ applied as urea-based fertilizer in both Paddy and GCRPS; and (3): N3: for the Paddy, 135 kg N ${ha}^{-1}$ applied in split application rates of 81 and 54 kg N ${ha}^{-1}$ (1 June 2021 and 27 May 2022) as urea-based and topdressing fertilizer, respectively (3:2 base-topdressing ratio of urea). However, for GCRPS, 67.5 kg N ${ha}^{-1}$ as urea and chicken manure was applied as a basal application (1:1 basal application ratio of urea and manure). All treatments received the same amount of phosphorus (42 kg P_2_O_5_ ${ha}^{-1}$ as Ca(H_2_PO_4_)_2_) and potassium (29 kg K_2_O ${ha}^{-1}$ as KCl).

**Figure 11:**
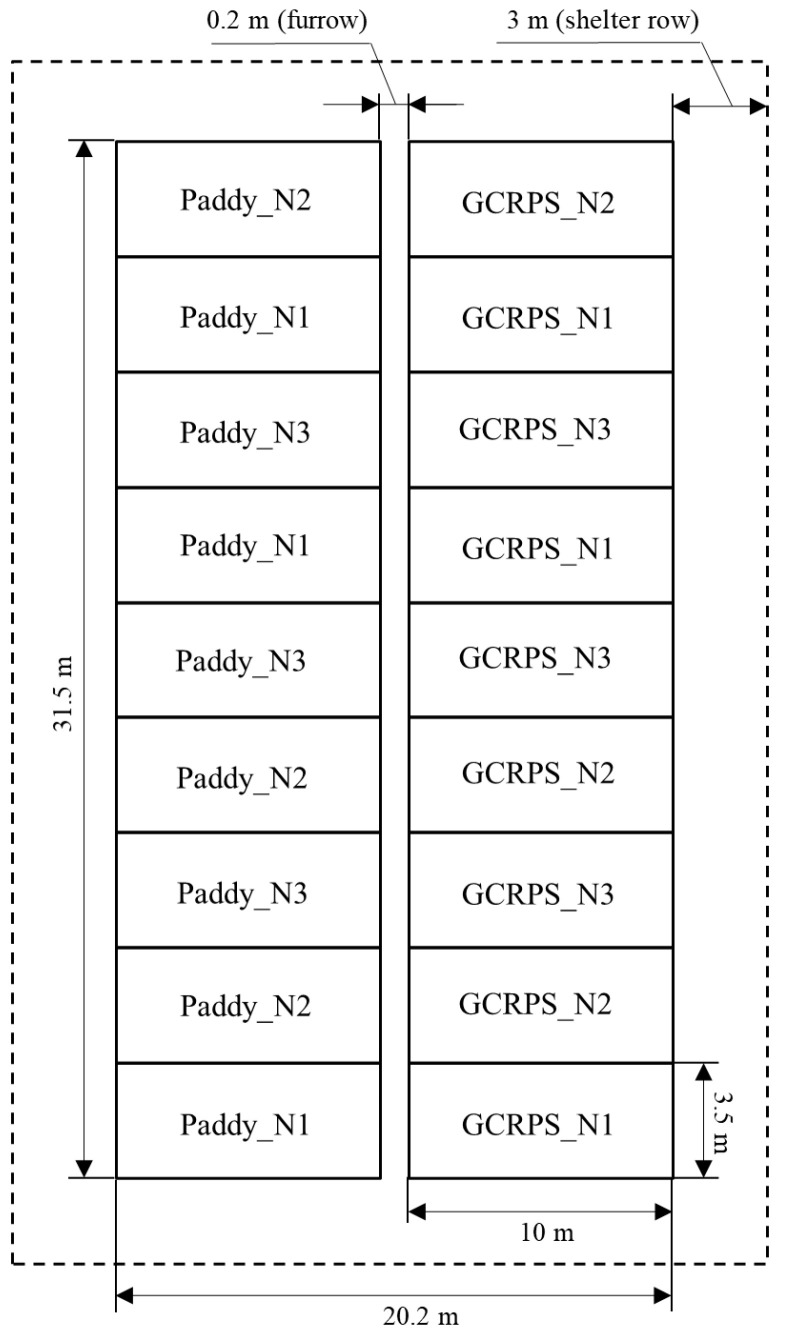
Diagram of plots. Paddy, traditional flooding paddy; GCRPS, ground cover rice production system; N1, no fertilizer; N2, urea-based fertilizer; N3, urea and manure-based fertilizer.

This field experiment comprised six experimental treatments, which involved combining two water treatments with three N treatments. Each treatment was replicated three times, resulting in a total of eighteen blocks measuring 3.5 m × 10 m each. The experimental field area spanned 20.2 m × 31.5 m, and shelter rows were established at a distance of approximately 3 m (Figure 11). In order to minimize the horizontal water and nutrient lateral flow between neighboring blocks, each block was constructed as a 0.3 m in width and 5 cm in depth ridge, with 50 cm of plastic film buried to separate them. The rice cultivar used in this experiment was ‘Chuankangyousimiao’, bred by the Sichuan Academy of Agricultural Sciences. Rice transplanting and harvesting in 2021 were performed on 19 May and 10 September and, in 2022, on 10 May and 31 August, respectively. The range of total irrigation in 2021 and 2022 was between 298 and 335 mm and 172 and 174 mm for the Paddy treatment and 65 mm and 38 mm for the GCRPS treatment, respectively (Table 4). The irrigation water source was underground well water, and flooding was used as the irrigation method. The quantity of water used for each irrigation was precisely measured using a water meter (GB/T778-96, Haiquan, Chengdu, China) [62]. Treatment in the Paddy was sprayed normally with herbicides and insecticides, while treatment in GCRPS was sprayed only at the panicle initiation stage because the plastic mulch could effectively suppress the occurrence of weeds and pests. Autumn plowing was not performed before the 2021 trial, but it was conducted before the 2022 trial, and the rest of the field management practices were consistent.

### 4.3. Observations and Measurement Methods

The soil water content, SMN dynamics, and crop growth indicators were measured during the experiment. Soil water content was measured via the drying method, and five points were selected in each block according to the “S” pattern. Soil samples were collected at 15-day intervals from 0 to 80 cm in each 20 cm layer. Each fresh soil sample was extracted with 1 mol L^−1^ KCl to determine the concentrations of mineral N using a continuous flow analyzer (AA3, Bran+Luebbe, Norderstedt, Germany).

The dynamics of tiller numbers during the growth period were recorded via a continuous observation of 10 plants at 10-day intervals beginning at transplant and ending at the grain filling stage. Plant height, LAI, and DM were measured at the stages of maximum tillering, panicle initiation, grain filling, and maturity. On each sampling date, 10 plants were harvested. Plant height was measured using a scale; LAI was measured manually using a ruler to measure the leaf length and width and was calculated as the length × width × 0.75; DM was oven-dried at 80 °C to a constant weight for determination. Meanwhile, the crop yield components were measured in 2022, and eight plants were randomly selected in each block to determine three factors of the yield before the final harvest and obtain PT, SP, and TGW. After each block was fully harvested, the seeds were dried and weighed to determine the crop yield.

The weather data at the experimental site were collected using a small automatic weather station. This station recorded real-time information on various elements, including air temperature, rainfall, wind speed, sunshine hours, and relative humidity.

### 4.4. Statistical Analysis

Excel 2018 (Microsoft Corporation, Redmond, WA, USA) was used for data processing. Graphs were generated using Origin 2018 (OriginLab Corporation, Northampton, MA, USA). An analysis of variance was conducted using SPSS 23 (International Business Machines Corporation, Armonk, NY, USA), and the significance of differences was tested using the F-test and least squares (LSD). Amos 26.0 (International Business Machines Corporation, Armonk, NY, USA) was adopted for SEM construction and analysis.

## 5. Conclusions

Compared with the Paddy, the effects of GCRPS on soil water and N dynamics in the hilly purple soil zone in the middle reaches of the Yangtze River were not significant. In 2021, in the absence of drought, early, low-temperature stress, and frequent rainfall, GCRPS suppressed crop growth to some extent, and tiller numbers, the LAI and DM were reduced by 1.1% to 31.6%, 0.6% to 20.4% and 4.4% to 18.8%, respectively. Compared to the Paddy, the yields of treatments N2 and N3 for GCRPS significantly reduced by 15.0% and 22.0% in 2021. However, in 2022, when there was drought, early low-temperature stress, and low rainfall, GCRPS promoted crop growth, and its tiller numbers, LAI and DM significantly increased by 13.7% to 115.4%, 17.3% to 81.0%, and 9.0% to 62.6%, respectively. The crop yield for GCRPS increased by 2.9% to 9.2% compared to the Paddy, indicating that GCRPS is suitable for the region with early low-temperature and dry years with poor rainfall.

In 2022, GCRPS significantly affected tiller numbers, plant height, LAI, DM, and PT, but not SP, TGW, crop yield, soil water, and N dynamics. GCRPS indirectly affected crop yield by significantly affecting tiller numbers and PT, respectively, indicating that tiller numbers and PT are the key drivers of crop yield under GCRPS.

The successful implementation of GCRPS relies mainly on effective water management. In a future study, it is crucial to avoid excessive water in GCRPS rice fields. By ensuring strict drainage practices in GCRPS during wet years, this technology can truly become a stable and authentic solution for increasing yields in regions without climate stress.

## Figures and Tables

**Table 1:** Analysis of variance F-statistics to assess the effects of water and N management practices and year on the crop yield.

Source	W	N	Y	W × N	W × Y	Y × N	W × N × Y
DF	1	2	1	2	1	2	2
F value	8.6 **	82.3 ***	2.0 ns	3.0 ns	26.2 **	15.7 **	1.3 ns

DF, degree of freedom; ns, not significant; ** or *** significant at 0.01 or 0.001 probability level; W, water management practice; N, N management practice; Y, year.

**Table 2:** Correlation of rice tiller numbers and accumulated rainfall at the tillering stage under different water management practices from 2021 to 2022 (n = 7).

Treatment	2021	2022
Paddy	0.73 ns	0.97 **
GCRPS	0.74 ns	0.96 **

ns, not significant; ** significant at 0.01 probability level.

**Table 3:** Physical and chemical properties of the surface soil layer (0–20 cm) at the experimental site.

pH	$\theta_{s}\;({cm}^{3}\;{cm}^{-3})$	$\rho \;(g\;{cm}^{-3})$	Particle Fraction (%)			Total N	SOC	NH_4_^+^−N	NO_3_^−^−N	AP	AK
			Sand	Silt	Clay	$\left(g\;{kg}^{-1}\right)$		$\left(\mathrm{mg}\;{kg}^{-1}\right)$			
7.73	0.6	0.97	18	51	31	2.3	66.6	56.2	16.3	14.7	55

*θ_s_* is the saturated water content; *ρ* is bulk density; SOC is soil organic matter; AP is available phosphorus; AK is available potassium.

**Table 4:** Total amount of irrigation (mm) for each treatment.

Year	Paddy_N1	Paddy_N2	Paddy_N3	GCRPS_N1	GCRPS_N2	GCRPS_N3
2021	335	316	298	65	65	65
2022	173	172	174	38	38	38

## Data Availability

Data are available on request due to restrictions, e.g., privacy-based or ethical.
